# Effectiveness of Capacity-Building and Quality Improvement Interventions to Improve Day-of-Birth Care in Kinshasa, Democratic Republic of the Congo

**DOI:** 10.9745/GHSP-D-23-00236

**Published:** 2024-02-28

**Authors:** Virgile Kikaya, Fernand Katembwe, Jacky Yabili, Marcel Mbwanya, Elana Dhuse, Patricia Gomez, Rachel Waxman, Diwakar Mohan, Hannah Tappis

**Affiliations:** a Jhpiego, Kinshasa, Democratic Republic of the Congo.; b Jhpiego, Baltimore, MD, USA.; c Department of International Health, Johns Hopkins Bloomberg School of Public Health, Baltimore, MD, USA.

## Abstract

In sub-Saharan African settings like the Democratic Republic of the Congo, high-quality care during childbirth and the immediate postpartum period is lacking in public facilities, necessitating multipronged interventions to improve care. We used a pre-post design to examine the effectiveness of a low-dose, high-frequency capacity-building and quality improvement (QI) intervention to improve care for women and newborns around the day of birth in 16 health facilities in Kinshasa, Democratic Republic of the Congo. Effectiveness was assessed based on changes in provider skills, key health indicators, and beneficiary satisfaction. To assess changes in the competency of the 188 providers participating in the intervention, we conducted objective structured clinical examinations on care for mothers and newborns on the day of birth, immediate postpartum family planning (PPFP) counseling and method provision, and postabortion care before and after implementation of training and at 6 and 12 months after training. Interrupted time series (ITS) analysis techniques were used to analyze routine health service data for changes in select maternal, newborn, and postpartum outcomes before and after the intervention. To assess changes in clients’ perceptions of care, 2 rounds of telephone surveys were administered. Before the intervention, less than 2% of participating providers demonstrated competency in skills. Immediately after training, more than 80% demonstrated competency, and 70% retained competency after 12 months. ITS analyses show the risk of early neonatal death declined significantly by 9% (95% confidence interval [CI]=4%, 13%, *P*<.001), and likelihood of immediate PPFP uptake increased significantly by 72% (95% CI=53%, 92%, *P*<.001). Client satisfaction improved by 58% over the life of the project. These findings build on previous studies documenting the effectiveness of clinical capacity-building and QI approaches. If implemented at scale, this approach has the potential to substantively contribute to improving maternal and perinatal health in similar settings.

## Abstract (precis)

A low-dose, high-frequency capacity-building approach coupled with quality improvement interventions improved health care provider performance and maternal and newborn health outcomes.

## Teaser

**Key Findings:**

- This study of 16 health facilities providing maternity care in Kinshasa, DRC, suggests that a low-dose, high-frequency capacity-building approach coupled with continuous mentoring and quality improvement interventions is effective in changing provider performance and improving quality of care and outcomes.
- Early neonatal death rates significantly decreased by 9% across all facilities, and client satisfaction, as measured by the mean person-centered maternity care score, improved by 58%.
- At 12 months post-training, at least 75% of providers across all 16 facilities demonstrated competency in key skills around the day of birth. More than 80% demonstrated competency in the treatment of incomplete abortion and more than 90% in contraceptive implant insertion.

**Key Implications:**

- Use of integrated low-dose, high-frequency training sessions in combination with mentoring and quality improvement methods should be considered in both in-service training and preservice education.
- Close collaboration with the National Program for Reproductive Health of the Ministry of Health, including training of national trainers, provides a foundation for local ownership, sustainability, and its validation as an approved approach to conduct in-service capacity-building of providers.

## INTRODUCTION

The Democratic Republic of the Congo (DRC) has some of the world’s highest maternal and newborn mortality ratios.^1^ Maternal mortality ratio is estimated at 693 per 100,000 live births (2017), the newborn mortality rate at 26.8 deaths per 1,000 live births (2020), and the stillbirth rate at 27 per 1,000 total births (2019).^1^^–^^3^ Despite national commitments and ongoing efforts to reduce preventable mortality, the country is not on track to reach the Sustainable Development Goal targets for reduction of the maternal mortality ratio to less than 70 maternal deaths per 100,000 live births and the neonatal mortality rate to less than 12 newborn deaths per 1,000 live births, or the Every Newborn Action Plan’s target of less than 12 stillbirths per 1,000 total births by 2030.^4^^,^^5^

The juxtaposition of the DRC’s high antenatal care utilization (82.4%) and facility birth rates (85.2%), with 94.1% and 99.7% in Kinshasa, respectively,^6^ and high rates of maternal and newborn mortality suggest that quality of care around childbirth and the immediate postpartum period at health facilities must be considered as a contributing factor to the high mortality rates. The Plan National de Développement Sanitaire identifies low provider competency as a direct contributor to maternal deaths in the DRC.^7^ A study conducted in 6 health zones in North Kivu, a province in Eastern DRC, highlights gaps in the quality of services offered by clinicians.^8^ This study revealed an increase in hospital maternal deaths between 2009 and 2014, despite services being offered by qualified providers (doctors in 35.5% of cases, nurses in 47.2% of cases); 80% of deaths were caused by postpartum hemorrhage, eclampsia, and infections.^8^ An evaluation of health services conducted across the country showed that 16% of providers (21% in Kinshasa province) offering maternal and newborn care were trained in emergency obstetric care in the 24 months preceding the evaluation.^9^

Many factors influence the provision of high-quality care, including lack of adequate preservice education and opportunities for continuing professional development, as well as infrequent supportive supervision.^10^ In low- and middle-income countries, training is often used as a strategy to improve health care provider performance; however, studies show the effectiveness of training is highly variable.^11^ In the DRC, the Ministry of Health has a nationally validated multiweek training curriculum on basic emergency obstetric, newborn care, and family planning (FP) that was offered off-site and for a limited number of providers. Supervision is mostly conducted with checklists and does not focus on health outcomes or problem-solving. Evidence suggests that the choice of training methodology is critical to improvement in provider performance.^12^ Training on-site (at a provider’s workplace) and training that incorporates clinical practice are more effective than the traditional alternatives,^9^ and strategies that combine training with other approaches, such as quality improvement (QI), tend to result in greater benefits in provider performance than training alone.^13^

Strategies that combine training with other approaches, such as quality improvement, tend to result in greater benefits in provider performance than training alone.

The United Nations defines capacity-building as “the process of developing and strengthening the skills, instincts, abilities, processes and resources that organizations and communities need to survive, adapt, and thrive in a fast-changing world.”^14^ The World Health Organization identifies clinical mentorship as a key intervention to improve and sustain the provision of high-quality clinical care, especially in countries with no organized system of continuing professional development after initial preservice training.^15^ One example of a successful capacity-building and clinical mentorship model is the “low-dose, high-frequency” (LDHF) approach that has been proven effective in decreasing fresh stillbirth and early newborn deaths at health facilities in Uganda^16^ and Ghana.^17^ This approach includes on-site trainings of all maternity health care providers in a health facility in relatively short (4–5 days) sessions followed by repetitive practice on anatomic models and clients, supervised by peer practice coordinators, and regular on-site mentoring and follow-up by mentors/trainers via mobile phone. This contrasts with traditional trainings that gather a few providers from many sites in conference room-based trainings for several days up to several weeks, depending on the amount of material to be covered. Comprehensive courses on the package of emergency obstetric and newborn care frequently range from 14 to 28 days.

Similar LDHF interventions have been effective in reducing perinatal mortality among preterm and low birth weight babies in Kenya and Uganda^18^ and have shown promise for improving both maternal and newborn care practices in India.^19^ However, there are few studies documenting the design and impact of multifaceted capacity-building and QI interventions on both maternal and newborn health outcomes and none that we could identify that included immediate postpartum FP (PPFP) among practices and outcomes of interest.

This study aimed to evaluate the integrated LDHF capacity-building and QI intervention in improving service quality and outcomes on the day of birth at 16 health facilities in Kinshasa, DRC. Although various approaches to improve the quality of maternal and newborn care^20^ and PPFP^21^ have been carried out in DRC, to our knowledge, this is the first evaluation of an integrated approach to improve the capacity of health care providers to deliver maternal and newborn health services around the day of birth, including immediate PPFP services.

## METHODS

### Study Setting

Kinshasa province is divided into 35 health zones (32 urban, 3 rural), with an estimated 761 facilities reporting on services in the national health information system. Our project supported the implementation of the integrated LDHF capacity-building and QI intervention in 16 facilities as a proof of concept. To identify sites for project implementation and evaluation, we used the following selection criteria: facilities with maternity caseloads of at least 30 births per month, offering FP services (although not necessarily PPFP services as few facilities were offering this), and not currently participating in another program on maternal and newborn health and/or QI. Other considerations included geographic distribution and willingness of facility management to participate in the project. The 16 selected facilities are located in 9 health zones and reflect the diversity of facility types meeting inclusion criteria (Table 1). Selected facilities included 7 hospitals and 9 health centers and a balance between public and private facilities. For comparison, in the 2013 Demographic and Health Survey, respondents in Kinshasa reported that 51.9% of all deliveries took place in a public sector facility and 46.0% in a private facility.^22^ This analysis was neither powered nor designed to detect differential effects (heterogeneity) across the types of facilities.

**TABLE 1.** Facility and Provider Characteristics of Participating Facilities, Kinshasa, Democratic Republic of the Congo

**Health facility characteristics (N=16)**	**No. (%)**
Affiliation	
Public	7 (44)
Private or faith-based	9 (56)
Facility capacity	
Basic emergency obstetric and newborn care	7 (44)
Comprehensive emergency obstetric and newborn care (referral facility)	9 (56)
Service volume	
Low (less than 50 births per month)	8 (50)
High (more than 50 birth per month)	8 (50)
**Health care provider characteristics (N=188) **	
Sex	
Male	54 (29)
Female	134 (71)
Cadre	
Doctor	44 (23)
Midwife	37 (20)
Nurse	107 (57)
Years of work experience	
1–5 years	39 (21)
6–10 years	40 (21)
10+ years	109 (58)

The first wave of interventions was implemented in 8 facilities starting in November 2017. The remaining 8 facilities began the intervention in July 2018. Facilities were stratified by affiliation (public/private) and service volume and randomly assigned to either the first or second wave of implementation.

### Intervention

Before launching the intervention at the first group of facilities, 16 master mentors were selected from the Ministry of Health’s roster of master trainers and prepared to offer the on-site training, on-site and remote mentorship, and support for QI initiatives. All master mentors were read information about the study and then signed an informed consent form.

The integrated LDHF intervention comprised 2 main components on capacity-building and QI. The capacity-building component included competency-based on-site training for teams of up to 15 providers (midwives, nurses, and doctors) with responsibility for the care of mothers and newborns on the day of birth at each facility, peer-supported skills practice, clinical mentoring via telephone calls, and site visits from master trainers. Because this was a package of basic interventions, all members of the team were able to provide all components. The QI component included establishing on-site QI teams, providing teams with technical support to develop and implement QI plans, and coaching teams on using data to monitor and drive QI efforts. An example of a QI aim is: “By the end of December 2018, 95% of partograms will be correctly filled in during labor by maternity staff.” QI teams assigned specific providers to monitor partograph use and coach peers, and regular reviews were conducted to assess progress. After the initial introduction, the QI committees guided their own work.

As part of the capacity-building intervention and to facilitate implementation and ensure a common foundation for evaluation of the training and QI interventions, the project supported minor renovations to maternity wards at some sites to ensure availability of running water and privacy for women during labor and birth. During the study period, the project also provided sites with equipment, such as delivery kits, equipment for immediate newborn care, and regular supplies of medications and consumables. All facilities also received a set of anatomic models (MamaNatalie, NeoNatalie, Sister U, RITA arm) to facilitate practice between training sessions.

The competency-based on-site training curriculum included 4 modules: (1) day-of-birth care for the mother-baby dyad, (2) immediate PPFP (before the woman is discharged from the facility after delivery) counseling and method provision, (3) postabortion care, and (4) management of low birth weight newborns and maternal and newborn infection (Table 2).

**TABLE 2.** On-Site Competency-Based Training Module Content for Capacity-Building and Quality Improvement Interventions to Improve Day-of-Birth Care

**Module 1:** Care for mothers and newborns around the day of birth	Support for normal labor and birth (respectful maternity care, infection prevention, use of partograph, active management of the third stage of labor, immediate newborn care, postnatal care) Treatment of postpartum hemorrhage Treatment of severe pre-eclampsia/eclampsia with magnesium sulfate Newborn resuscitation Consistent recordkeeping, use of data for decision-making and quality monitoring
**Module 2:** Immediate postpartum FP counseling and clinical skills	FP counseling in the antenatal and postpartum periods on all methods available for immediate postpartum use Clinical skills for postpartum FP provision, including side effect management and removal (of intrauterine devices and implants) Youth-friendly FP services for postpartum and postabortion clients
**Module 3:** Postabortion care	Treatment of incomplete abortion using manual vacuum aspiration and misoprostol Postabortion FP counseling Clinical skills for postabortion FP provision, including side effect management and removal Identification and/or referral for additional reproductive health services Youth-friendly postabortion care services
**Module 4:** Management of the low birthweight newborn and management of newborn sepsis	Recall of newborn danger signs and their management Basic management of low birthweight newborns, including kangaroo mother care Antibiotic regimens for women with infection and newborns with possible severe bacterial infections How to establish and manage kangaroo mother care units
**Cross-cutting elements addressed in all modules and clinical practice**	Respectful maternity care Infection prevention and control Clinical decision-making Consistent recordkeeping, use of data for decision-making and quality monitoring using dashboards

Abbreviations: FP, family planning.

Each module consisted of on-site training (4–5 days) facilitated by a pair of master mentors. Modules 1–3 included pre- and post-training knowledge and skills assessments using objective structured clinical examinations (OSCEs). Trainings were capped at 15 maternity, FP, and/or pediatric service providers per site, with priority given to maternity ward staff. This number was determined to ensure appropriate trainer-to-participant ratios and an optimal learning environment. For hospitals, this was only a portion (30%–60%) of all eligible staff, while at the health centers, the training cohorts ranged from 9–11 participants and included all eligible providers. Across the 16 intervention sites, 188 providers participated in the on-site trainings, mentoring, and QI interventions (Table 1). All providers were read information about the study and signed an informed consent form before the training sessions began.

The training curriculum was implemented over a 4-month period (1 module per month with 3 weeks of practice between modules). During the Module 1 training sessions, 2 participants per site were selected to serve as practice coordinators and provided with additional orientation on how to conduct clinical skills demonstrations, coach peers, and provide feedback on performance using standardized practice checklists. Between on-site training sessions, peer practice coordinators maintained regular contact with master mentors supporting their facility and ensured that all participating providers practiced skills covered during the training. Between on-site training sessions, master mentors also provided additional guidance and support to providers by phone or by visiting the facilities on an established schedule, with a focus on specific services or skills where providers felt uncomfortable and/or where OSCEs indicated a need for improvement.

QI teams were established at each facility and oriented to a 6-step approach developed by the Survive and Thrive Global Development Alliance to identify areas for improvement and prioritize solutions.^23^ QI team members were selected from among trained providers and other facility staff members (management, cleaning technicians, and pharmacists) and supported to develop quarterly improvement plans based on issues identified in each facility. Project staff visited sites regularly to support QI plan development, implementation, and evaluation processes and mentor facility staff in the use of data for decision-making and quality monitoring. Financial support (up to US$1,200 per facility per quarter) was provided by the project to support implementation of priority activities.

As Module 2 on PPFP was rolled out, the project team identified that FP counseling was not well integrated into antenatal care. To address this, training sessions specifically targeting antenatal care providers were organized to orient them to the key messages and methods for PPFP counseling, including intrauterine devices (IUDs), implants, progestin-only pills, and lactational amenorrhea. Tubal ligation was not included as there were no providers in the study facilities capable of offering the procedure.

### Study Design, Data Collection, and Analysis

The evaluation was based on the theory of change that postulated that improving the skills of providers would improve the technical quality of care provided to women and newborns during childbirth and experiential quality as measured by their perception of the care experience. We evaluated the effectiveness of the project through changes pre- and post-intervention in: (1) provider competency, measured by OSCEs and implementation of high-impact practices pre- and post-intervention; (2) select maternal and newborn health outcomes using an interrupted time series analysis of the routine health facility data; and (3) client perceptions of care and satisfaction with services using the modified Afulani scale, measured by telephone surveys with a subset of women who gave birth at project sites.

#### Competency Assessment

Providers in all facilities were assessed on 6 skills (support for normal labor and birth, manual removal of placenta, neonatal resuscitation, postpartum IUD insertion, single-rod contraceptive implant insertion, and removal of retained products of conception with manual vacuum aspiration) using OSCEs facilitated by master mentors immediately before and after implementation of each training module and at 6 and 12 months after training. OSCE results were collected using skills checklists while providers demonstrated their skills on anatomical models. The OSCE instruments included validated learner assessments of the Helping Mothers Survive module on Bleeding After Birth and the Helping Babies Survive module Helping Babies Breathe.^24^^,^^25^ Providers who correctly performed 80% of the items on the checklist were considered to have demonstrated competence in that skill. For all OSCEs, the individual provider was the unit of analysis. OSCE data from providers at all 16 facilities were pooled, and the proportion of providers scoring at least 80% on OSCEs at each time point is presented.

#### Routine Health Service Data

We collected routine service delivery statistics from the Ministry of Health-mandated maternity register and 3 supplementary registers (i.e., delivery register, postabortion care register, and pediatric admission register) introduced by the project to capture additional processes and outcomes of interest for this study at each facility. Providers were oriented on how to fill in project-specific registers and tools. Summary statistics (aggregate counts) were collected monthly from all 16 facilities over a 35-month period from August 2017 through June 2020. This provided approximately 3 months of pre-intervention and 32 months of post-intervention data for the 8 facilities where implementation started in November 2017 and 11 months of pre-intervention and 24 months of post-intervention data for the second group of facilities where implementation started in July 2018.

For outcomes of interest, such as fresh stillbirth rate, early neonatal death rate, and immediate PPFP uptake, the effect of the intervention was analyzed using an interrupted time series model. The month of training was considered as the “zero” month for the assessment of the change in outcomes, assessed using segmented regression models. To account for the differences in calendar time for implementation of the intervention, the time variable was centered on the month of implementation for each individual facility—time was negative before the month of intervention, decreasing by 1 unit going backward and increasing by 1 unit after the intervention. Segmented regression models were fitted with the monthly aggregate count of the outcome for each facility as the outcome variable. The objective of the analysis was to assess the effect of the intervention on the outcomes in the immediate period after intervention initiation (level change) and in the months after the intervention (post-intervention slope) while accounting for secular trends (pre-intervention slope). We assumed an immediate effect (no lag) would present in the next month following the intervention and fit a population-averaged generalized linear model estimate using generalized estimating equations with a Poisson distribution, log link, and exchangeable correlation. Given the high correlation at the facility level and the need to estimate the average effect, the primary analysis accounted for clustering within the facility using generalized estimating equations. The model included the time variable, dummy for implementation (0 before start of implementation and 1 after), and their interaction term, apart from facility-level characteristics, such as facility type (referral facility or not), baseline number of deliveries at the facilities (high and low), and facility ownership (public or private) as control variables. Incidence rate ratios from the Poisson generalized estimating equations model and their 95% confidence interval (CI) are presented. The estimate for the time variable is considered the pre-intervention slope, the interaction variable is the post-intervention slope, and the estimate for the implementation dummy is the level change.

We used descriptive statistics to compare indicators for the management of complications, such as proportion of women with severe pre-eclampsia/eclampsia receiving a loading dose of magnesium sulfate and newborns not breathing at birth that were successfully resuscitated. Because the number of complications was low and not commonly seen across all facilities for all time points (referral facilities and facilities with high volume are more likely to see clients experiencing complications), the indicators on the management of complications are analyzed and presented as pre-post estimates (before and after introduction of intervention at each of the facilities) aggregated over all included facilities.

#### Client Surveys

We assessed client perceptions of experience and satisfaction with care at 2 time points after the start of implementation of the first phase, in May 2018 (n=208 clients) and November 2019 (n=328 clients). Clients were recruited from among women who gave birth at the 16 facilities between May and June 2018 and November and December 2019, respectively. Clients were recruited during their stay in the maternity wards and participated in a telephone survey during the month following their discharge from the health facilities. Clients who were recruited to participate in the telephone survey were read information about the study and provided written consent when enrolled before discharge from the facility after childbirth. Each participant had the option to leave the study at any time. We conducted a structured interview using a validated 30-item scale developed by Afulani et al. to measure person-centered maternity care with 3 subscales: dignified and respectful care, communication and autonomy, and supportive care.^26^ We analyzed changes in person-centered maternity care using the condensed 13-item version of the scale.^27^ We calculated the mean score for the 13 items on a scale of 0–3 items for the 2 different time points. Using linear regression accounting for clustering at the facility level, we calculated the change in mean scale scores from the pre-intervention to the post-intervention survey, adjusting for the facility’s phase of implementation, type of facility, and whether the facility was the nearest to home as reported by the respondent. All analyses were done using Stata version 15.0.

### Ethical Approval

This study received ethical approval from the Institutional Review Board of the Johns Hopkins Bloomberg School of Public Health (IRB No. 8279) and from the Ethics Committee of the Kinshasa School of Public Health (ESP/CE/072/2017). The study was also registered on clinicaltrials.gov (https://clinicaltrials.gov/ct2/show/NCT03363308).

## RESULTS

Across the 16 facilities, average baseline scores on OSCEs were low. Before the intervention, providers, on average, correctly demonstrated only 32% (95% CI=30%, 35%) of expected steps in supporting normal labor and birth, 35% (95% CI=31%, 37%) of expected steps for manual removal of placenta, and 28% (95% CI=25%, 30%) of steps for newborn resuscitation, which were part of the first training module. For the second and third modules, providers correctly performed only 5% of the tasks (95% CI=3%, 7%) for postpartum IUD insertion, 12% (95% CI=8%, 15%) for single-rod contraceptive implant insertion, and 7% (95%=CI 4%, 10%) for treatment of incomplete abortion with manual vacuum aspiration before the start of each module. Only 8 of 188 providers (<2%) demonstrated competency in any of these essential skills with a score of at least 80% on any of the baseline OSCEs (Table 3).

**TABLE 3.** Providers Scoring at Least 80% on OSCE on Essential Skills for Providing Quality Care on the Day of Birth

	**N=188 Providers at 16 Project Sites**			
**Skill**	**Pre-Training,** **No. (%)**	**Immediately Post-Training,** **No. (%)**	**6 Months Post-Training,** **No. (%)**	**12 Months Post-Training,** **No. (%)**
**Module 1: Care for mothers and newborns on the day of birth**				
Support for normal labor and birth	2 (1.1)	152 (83.1)	135 (77.1)	142 (84.5)
Manual removal of placenta	1 (0.5)	152 (83.1)	151 (86.3)	155 (92.3)
Newborn resuscitation	0 (0.0)	147 (80.3)	99 (56.6)	126 (75.0)
**Module 2: Immediate postpartum family planning counseling and clinical skills**				
Postpartum intrauterine device insertion	0 (0.0)	133 (74.7)	137 (83.5)	121 (79.1)
Contraceptive implant insertion (single rod)	2 (1.1)	157 (88.2)	138 (84.7)	138 (90.2)
**Module 3: Postabortion care**				
Treatment of incomplete abortion with manual vacuum aspiration	3 (1.6)	150 (82.4)	133 (78.7)	135 (87.7)

Abbreviation: OSCE, objective structured clinical examination.

### Improvements in Health Care Provider Competency and Service Provision

The proportion of providers scoring at least 80% on OSCEs immediately following completion of each module and at 6 and 12 months post-training was significantly higher. At least 3 in 4 providers demonstrated competency with OSCE scores of at least 80% immediately after completion of each module. For postpartum IUD insertion, 74.7% of participants scored at least 80% immediately after training. At 6 months post-training, the proportion demonstrating competency in some skills (i.e., manual removal of placenta, postpartum IUD insertion, postpartum contraceptive implant insertion) increased slightly, while the proportion demonstrating competency in other skills (i.e., support for normal labor and birth, treatment of incomplete abortion with manual vacuum aspiration*)* declined slightly and newborn resuscitation declined more notably. At 12 months post-training, following further mentorship, peer practice, and QI support, at least 75% of providers across all 16 facilities demonstrated competency in essential skills for providing quality care around the day of birth. More than 80% demonstrated competency in support for normal labor and birth and treatment of incomplete abortion with manual vacuum aspiration, and more than 90% in manual removal of placenta and implant insertion.

At 12 months post-training, following further mentorship, peer practice, and QI support, at least 75% of providers across all 16 facilities demonstrated competency in essential skills for providing quality care around the day of birth.

Improved competency was also evident in the implementation of high-impact practices across all 16 facilities. For example, at the time of the first training module, 21.7% (5 of 23) of women with severe pre-eclampsia or eclampsia received a loading dose of magnesium sulfate, compared to 97.4% (37 of 39) in the 6 months post-training. Similarly, the proportion of newborns not breathing at birth that were successfully resuscitated increased from 56.3% (18 of 32) during the month of training of the first training module compared to 95.1% (79 of 82) in the 6 months post-training. There were no differences in the change in scores by type of facility or the volume of utilization. All providers showed improvement in scores at 6 and 12 months post-training. Supplement Figure S1 provides results by facility type.

### Improvements in Outcomes

The baseline level of PPFP adoption was very low due to the lack of provider knowledge and unavailability of commodities at many facilities. Trends in immediate PPFP adoption showed a constant increase from the start of the intervention (Figure).

**FIGURE:**
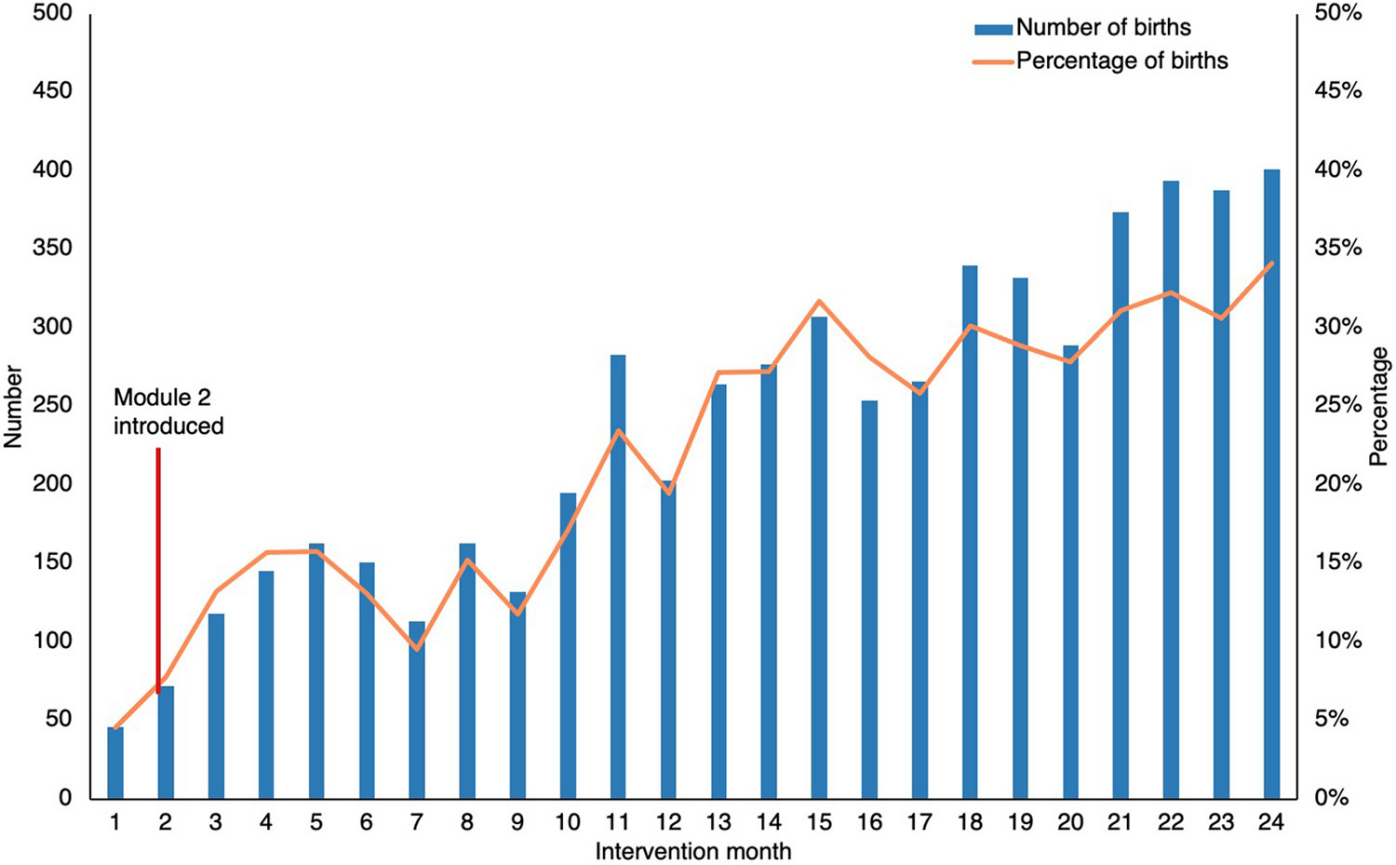
Number and Percentage of Births With Women Adopting a Modern Contraceptive Method Before Discharge From Health Facility After Childbirth, Kinshasa, Democratic Republic of the Congo

The segmented regression models for key outcomes of interest from the service statistics adjusted for size and type of facility and phases of program implementation are summarized in Table 4. Before the intervention, early neonatal death rates (slope: 1.07, 95% CI=1.06, 1.07) and immediate PPFP uptake (slope: 1.36, 95% CI=1.31, 1.40) showed statistically significant positive slope (rising trends), while the fresh stillbirth rate and postpartum hemorrhage rate appeared stable (no significant slope). Immediately after the intervention (level change), the risk of fresh stillbirth decreased by 59% (95% CI=1, 83), but the estimates did not achieve statistical significance, while early neonatal death rates significantly decreased by 9% (95% CI=4, 13). Immediate PPFP uptake increased significantly by 72% (95% CI=53, 92), while rates of postpartum hemorrhage remained unchanged.

**TABLE 4.** Improvements in Client Outcomes After Capacity-Building and Quality Improvement Interventions

	**Client Outcomes, N=550**											
	**Fresh Stillbirth**			**Early Neonatal Death**			**Immediate PPFP Uptake**			**Postpartum Hemorrhage**		
	IRR^a^	*P* Value	95% CI	IRR^a^	*P* Value	95% CI	IRR^a^	*P* Value	95% CI	IRR^a^	*P* Value	95% CI
Level change due to intervention	0.41	.05	0.17, 1.01	0.91	<.001	0.87, 0.96	1.72	<.001	1.53, 1.92	1.01	.83	0.96, 1.05
Pre-intervention slope	0.99	.80	0.88, 1.10	1.07	<.001	1.06, 1.07	1.36	<.001	1.31, 1.40	1.00	.36	1.00, 1.01
Post-intervention slope	1.08	.19	0.96, 1.21	0.94	<.001	0.93, 0.94	0.77	<.001	0.74, 0.79	1.00	.38	0.99, 1.00

Abbreviations: CI, confidence interval; IRR, incidence rate ratio; PPFP, postpartum family planning.

^a^ Adjusted for size and type of facility, phases of program implementation.

In the months immediately following the intervention, the early neonatal death rate and immediate PPFP uptake showed statistically significant decreasing trends (Table 4).

As with the OSCE scores, there was no difference in client outcomes between private and public facilities or low-volume and high-volume facilities. We have sought to describe the effects between private and public facilities visually as an exploratory subgroup analysis in Supplement Figures S1 and S2.

### Improvements in Client Experience and Satisfaction With Care

Differences in client experience and satisfaction with care reported in the May 2018 and November 2019 surveys are presented in the Supplement Table. On a scale of 0–3, we saw an increase of 58% (95% CI=50%, 68%) in the mean person-centered maternity care score after adjusting for the phase of implementation, type of facility, and whether the facility was the nearest from home for the respondent. Similar increases were observed in all 3 subscales (dignified and respectful care, communication and autonomy, and supportive care), with positive changes documented in all 13 scale items (Supplement Table). Women who reported being provided the choice of laboring position were low across both rounds.

## DISCUSSION

To our knowledge, our evaluation is the first of its kind for a multipronged intervention of the type carried out in the DRC. The integrated LDHF capacity-building and QI intervention was effective in improving quality of care and outcomes on the day of birth at the 16 intervention sites in Kinshasa, DRC. Whereas less than 2% of providers successfully demonstrated competency in the 6 skills assessed before training, at 12 months after training, 75% were competent in these skills. Risks of fresh stillbirth and early neonatal death decreased, likelihood of immediate uptake of PPFP increased, and validated measures of client experience of care improved.

To our knowledge, our evaluation is the first of its kind for a multipronged intervention of the type carried out in the DRC.

More than 80% of providers passed the OSCEs immediately after training, with the exception of postpartum IUD insertion, which was a new skill for both master mentors and participants. Maintenance of newly acquired skills over time is a well-documented challenge.^28^ Observed declines in OSCE scores over the 12 months post-training may be explained by reversion to normative practices, as frequency of practice sessions and use of checklists to monitor skills decreased. Programmatic efforts to mitigate these declines and facilitate skill retention included site visits to encourage maintenance of practice sessions and provision of certificates attesting to provider competence at 12 months post-training. At 12 months, over 80% of providers scored above 80% on all but 2 competencies (newborn resuscitation and postpartum IUD insertion). Considering that some providers passed with very high scores and those who did not pass were close to 80%, analysis suggests that competencies, even if they are low initially, can be improved and maintained with an appropriate LDHF approach and emphasis on on-site practice and mentorship. This is important because in the DRC in general, and particularly in Kinshasa, capacity-building for maternal, newborn, and child health and FP is conducted through long, off-site workshops without systematic follow-up and skills measurement.

This study also provides evidence of the effectiveness of the combination of LDHF capacity-building, QI, support, and resource provision in reducing the risk of fresh stillbirth and neonatal death in the context of the health system in Kinshasa. The reduction in fresh stillbirth is likely due to 2 major factors: (1) improved care during labor and birth, including use of the partograph for regular monitoring of the woman and fetus and rapid identification and treatment of complications, as noted by Hofmeyr et al.^29^; and (2) resuscitation of all non-macerated babies not breathing/crying at birth. The reduction in early neonatal death can also be explained by improved intrapartum care, leading to fewer asphyxiated babies, and improved knowledge and skills in newborn resuscitation that led to improved survival of those babies requiring resuscitation. These findings are consistent with other studies that have looked at competency-based trainings with measurement of health outcomes.^16^^,^^30^^,^^31^ The findings show that even in very low-resource health settings like the DRC, evidence-based capacity-building interventions can avert intrapartum-related neonatal deaths when preconditions for service provision (adequate supplies, medicines, and working conditions) are met.^32^

In the DRC, facilities are considered to have integrated FP services when they have at least 2 trained FP providers and offer at least 3 FP methods. Pre-intervention OSCE results suggest that, by this definition, having integrated FP services may not be a reliable indication of facility capacity to provide immediate PPFP. Over the life of the project, the likelihood of immediate PPFP uptake increased significantly—by the end of the project, nearly 35% of women were adopting an FP method before being discharged from the hospital. The results indicate that a notable portion of women will use PPFP services if they are counseled and offered a method at this crucial time (before leaving the facility after delivery).

The interventions implemented in this study included a large range of clinical services that are offered around the day of birth and immediate postpartum period. The capacity-building approach targeted support of normal labor and birth, prevention/management of complications, provision of FP services, postabortion care, and management of maternal and newborn infection. This integrated, holistic model considered several high-impact competencies that should be offered by the team of maternity providers. Also, the combination of on-site training sessions and mentorship visits has allowed a quick improvement with minimal disruption to services. Mentors were able to assist the newly trained team to improve site set-up while offering them adequate support. In some instances, mentors helped put in place new materials and equipment that were stored at the facility.

This intervention broke up a traditional training of 21–28 days covering priority maternal and newborn interventions into smaller “bites” that were accessible to all relevant providers and fit facilities’ needs and schedules—thus, “low dose” compared to traditional training. The use of regular practice sessions in each facility at times convenient to them constituted the “high frequency” component. With on-site training, costs are lower than off-site workshops while also reaching all staff in that facility so that they can perform as a team. Lodging, long-distance transport, and full catering are not needed. Providing lunch or a coffee break/snack and local transport as motivation keep costs down. Because nearly all relevant providers are trained at the same time, new interventions are incorporated more rapidly using a team-oriented approach.

At the time of this intervention, online learning support was not available. However, with advances in the use of online resources via smartphones, it would be feasible to incorporate this into an LDHF approach.

Implementation of QI activities, facility infrastructure improvement, and providers’ improved competency in patient-centered care may have contributed to observed improvements in client experiences and satisfaction with care. Checklists contained respectful maternity care elements and were utilized during regular practice sessions and with clients. Results have shown that rapid improvement in providers’ capacity to improve services is possible even in low-resource settings and that it can be sustained over time. Considering the providers’ competency at baseline, a well-designed capacity approach such as this one can contribute to improving the quality of health services.

A key aspect of the intervention was the local ownership, which is important for sustainability. Master mentors who supported the intervention were recognized clinicians and national trainers, and the National Program for Reproductive Health, which is responsible for reproductive health programming, co-steered implementation, allowing the Ministry of Health to participate in the contextualization process and review challenges and successes during implementation. Shortly after the end of the project, the package of LDHF and QI materials was validated by the Ministry of Health as a nationally approved in-service training approach.

A key aspect of the intervention was the local ownership, which is important for sustainability.

A strength of this study is its assessment of the key pillars of the Donabedian framework—processes (provider competence) and outcomes. Through a heterogenous sample of facilities, it demonstrates potential generalizability to other similar settings in sub-Saharan Africa. Urban facilities providing emergency obstetric care, where the third delay (timely provision of quality care at the facility) is most important, can use the evidence for the effectiveness of LDHF training and QI activities on outcomes.

### Limitations

Limitations for assessing the causal link from intervention to effect in the study include the inability to separate effects due to multiple program components and accurately predict the start of improvements after the start of program implementation. It may be that providers modified behaviors due to the focus on their facilities by the study and implementing personnel. Implementation of multiple components happened over many months, which makes it difficult to pinpoint an exact time of change (and there may not be one) post-intervention. Other studies have identified response bias as a challenge in obtaining accurate insights into client perceptions and experiences of care.^33^ While interviewers clearly identified themselves as not being linked to the facilities, responses may not be free of social desirability. However, any such bias is likely to be prevalent at both rounds of the survey and may not account for the increase in mean scores. Selection of intervention facilities did not occur randomly and is subject to preexisting baseline characteristics and/or political exigencies. This makes it difficult to account for selection biases due to differences in baseline characteristics that may influence outcomes, making attribution to the program activities more challenging. The sample size of 16 facilities may have limited power to detect small changes either due to the program or a secular trend.

## CONCLUSION

Despite methodological limitations common to multifaceted health program evaluations in real-world implementation settings, this study suggests that the LDHF capacity-building approach coupled with QI interventions is effective in changing provider performance and improving health outcomes in Kinshasa, where the facilities are underfunded and understaffed, providers are poorly paid, and the infrastructure and equipment are in poor condition.

The current intervention focused on LDHF capacity-building coupled with a QI process. When introducing the LDHF approach, it is important to consider other aspects of QI beyond service providers, including the leadership and management capacity of administrative decision-making units (such as districts) and health facilities, supply chain and drug management issues, and other components that will reinforce the overall quality of services at the site.

In line with the World Health Organization standards for improving the quality of maternal and newborn care in health facilities,^34^ the DRC needs to promote models where health providers are regularly receiving in-service training on specific competencies and where the physical environment and quality of services are consistently monitored and improved. With LDHF as designed in this study, facilities are in a position to conduct refreshers on site and identify and propose solutions for service gaps. Innovations to complement the use of LDHF include web-based courses and resources that providers can access pre- and post-training. LDHF can also be incorporated as discrete modules into preservice education for midwives, nurses, and doctors and has the advantage of being focused on practicing clinical competencies, something that is a challenge for many students.

Introducing LDHF coupled with QI at large scale in the DRC is an opportunity that can change maternal and newborn health outcomes and improve the modern contraceptive prevalence rate. As the Ministry of Health is embarking on promoting the scale-up of this approach, adaptations to the model might need to be designed for rural DRC settings where health centers are remote from the referral hospital and have more difficult access to master mentors and support. Scale-up of the intervention is being implemented through current, large maternal health and family planning donor-funded projects. For each program, the intervention has been adapted to meet project needs (additional technical elements, module adaptation, and adjustments for rural provinces). The most important opportunity during scale-up is building the capacity of provincial levels to offer adequate training and mentorship. This allows more targeted local intervention and improvements. Another question that will be answered in a subsequent scale-up is the cost associated with the intervention. The cost will depend on how the approach is adapted in various models. The scale-up will be closely monitored and results communicated.

## Supplementary Material

GHSP-D-23-00236-Supplement.pdf

## Notes

**Peer Reviewed**

**First published online:** February 17, 2024
