# Supplementary material for: Effectiveness of Capacity-Building and Quality Improvement Interventions to Improve Day-of-Birth Care in Kinshasa, Democratic Republic of the Congo
Source: Glob Health Sci Pract. 2024 Feb 28;12(1):e2300236. doi: 10.9745/GHSP-D-23-00236 (PMC10906559; doi:10.9745/GHSP-D-23-00236)
Supplement: GHSP-D-23-00236-Supplement.pdf [file GHSP-D-23-00236-Supplement.pdf]

Supplement to: Kikaya V, Katembwe F, Yabili J, et al. Effectiveness of capacity-building and quality improvement interventions to improve day-of-birth care in Kinshasa, Democratic Republic of the Congo. *Glob Health Sci Pract*. 2024;12(1):e2300236. https://doi.org/10.9745/GHSP-D-23-00236

FIGURE S1. OSCE Scores by Facility Type (Volume, Public/Private)

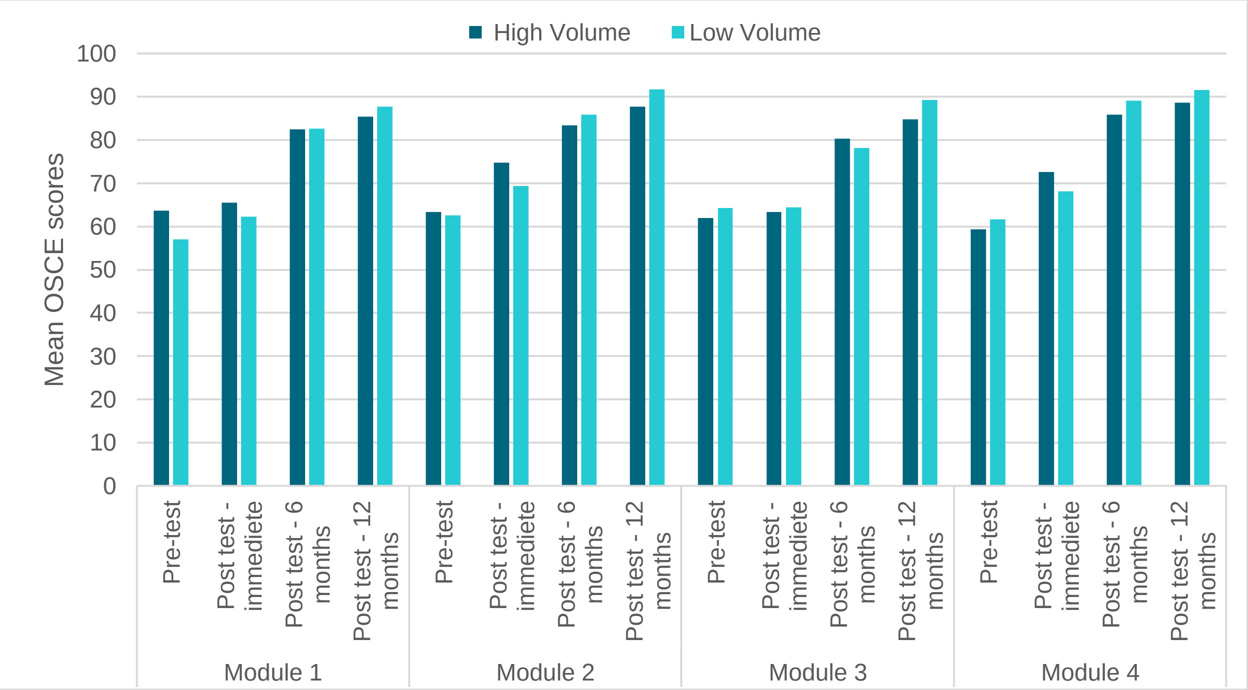

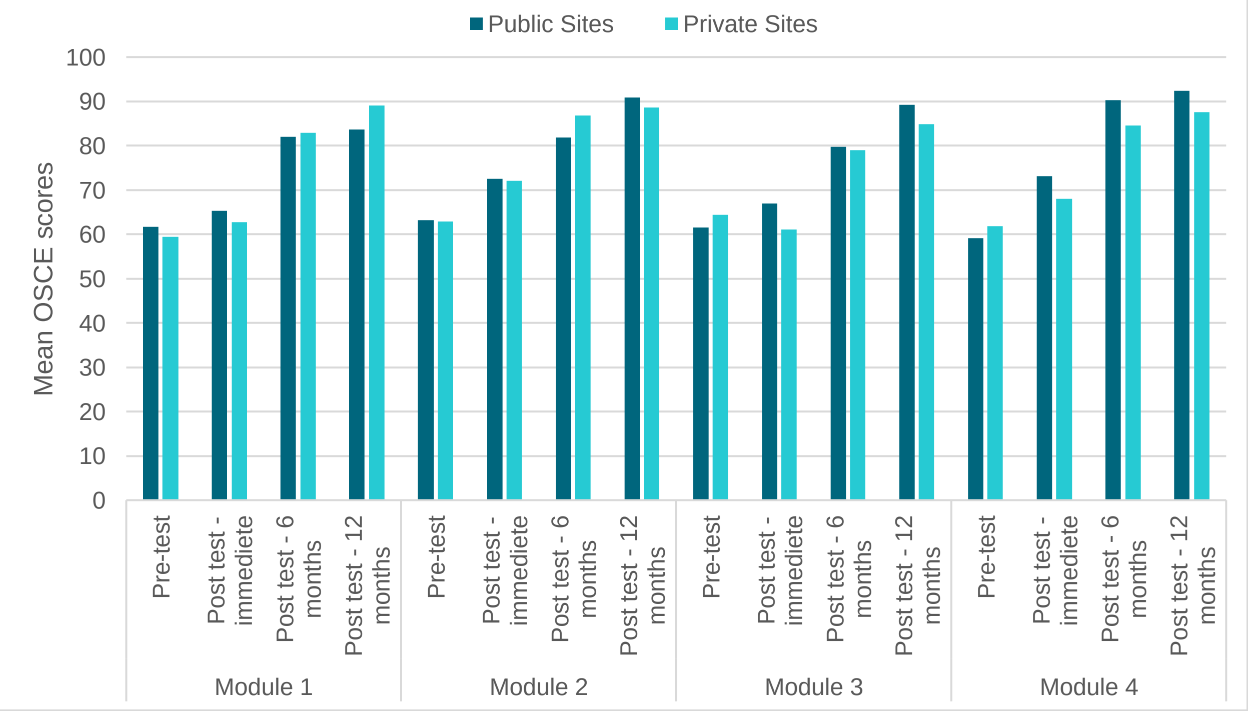

Supplement to: Kikaya V, Katembwe F, Yabili J, et al. Effectiveness of capacity-building and quality improvement interventions to improve day-of-birth care in Kinshasa, Democratic Republic of the Congo. *Glob Health Sci Pract*. 2024;12(1):e2300236. https://doi.org/10.9745/GHSP-D-23-00236

FIGURE S2. Service Delivery Outcomes by Facility Type

A. Public vs. Private Facilities

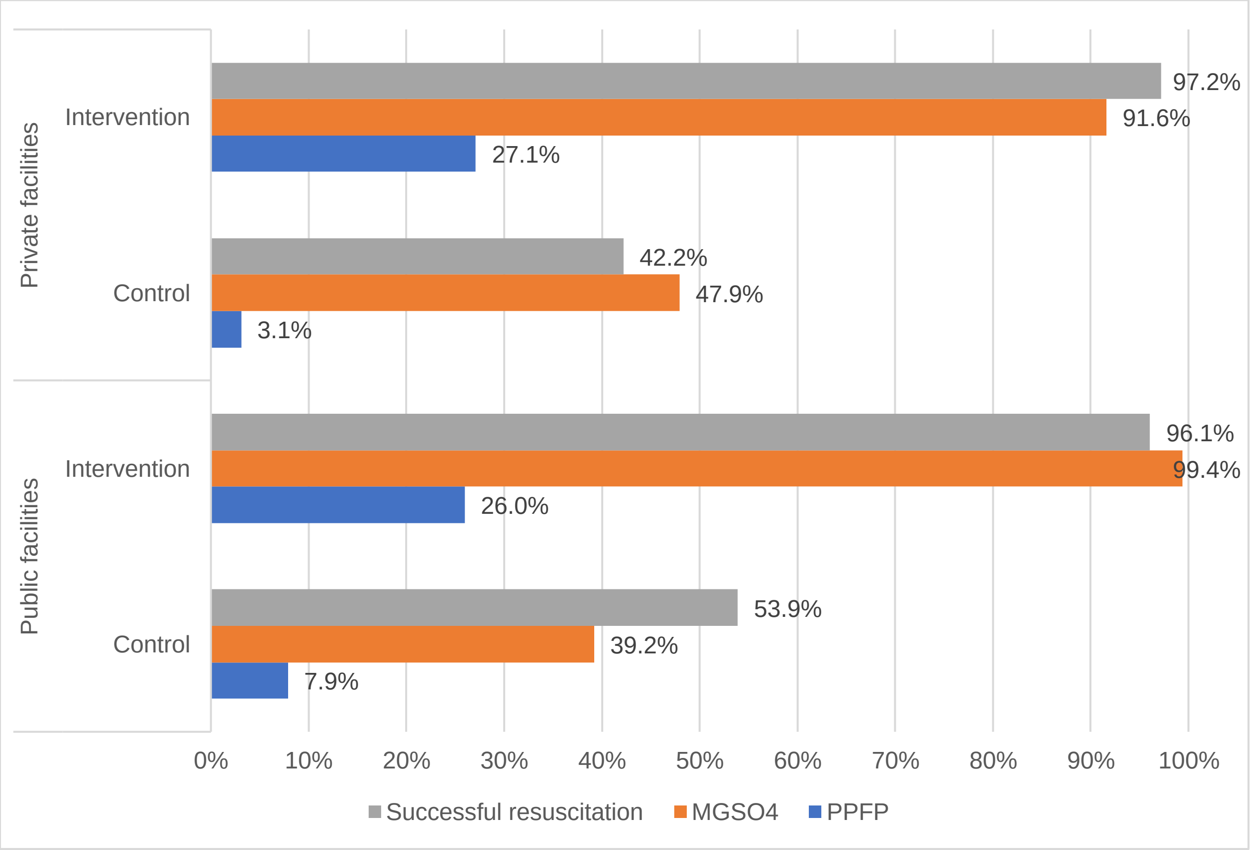

B.

Supplement to: Kikaya V, Katembwe F, Yabili J, et al. Effectiveness of capacity-building and quality improvement interventions to improve day-of-birth care in Kinshasa, Democratic Republic of the Congo. *Glob Health Sci Pract*. 2024;12(1):e2300236. https://doi.org/10.9745/GHSP-D-23-00236

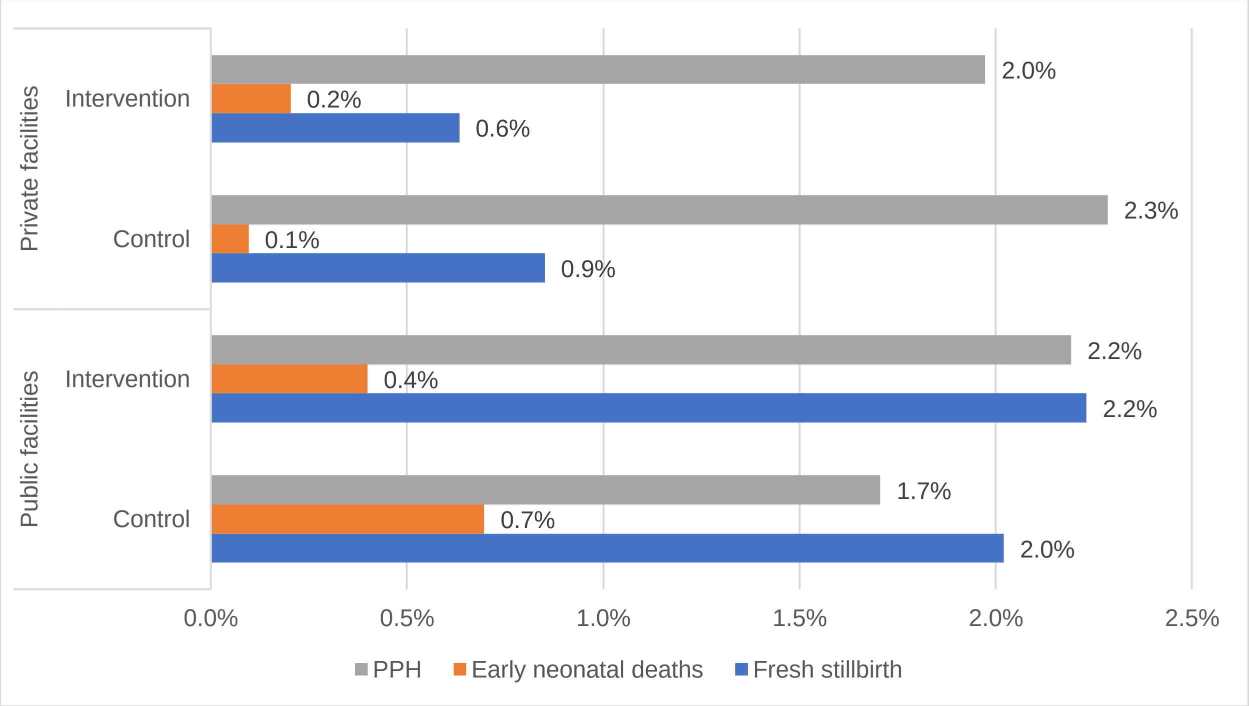

C. High Volume vs. Low Volume Sites

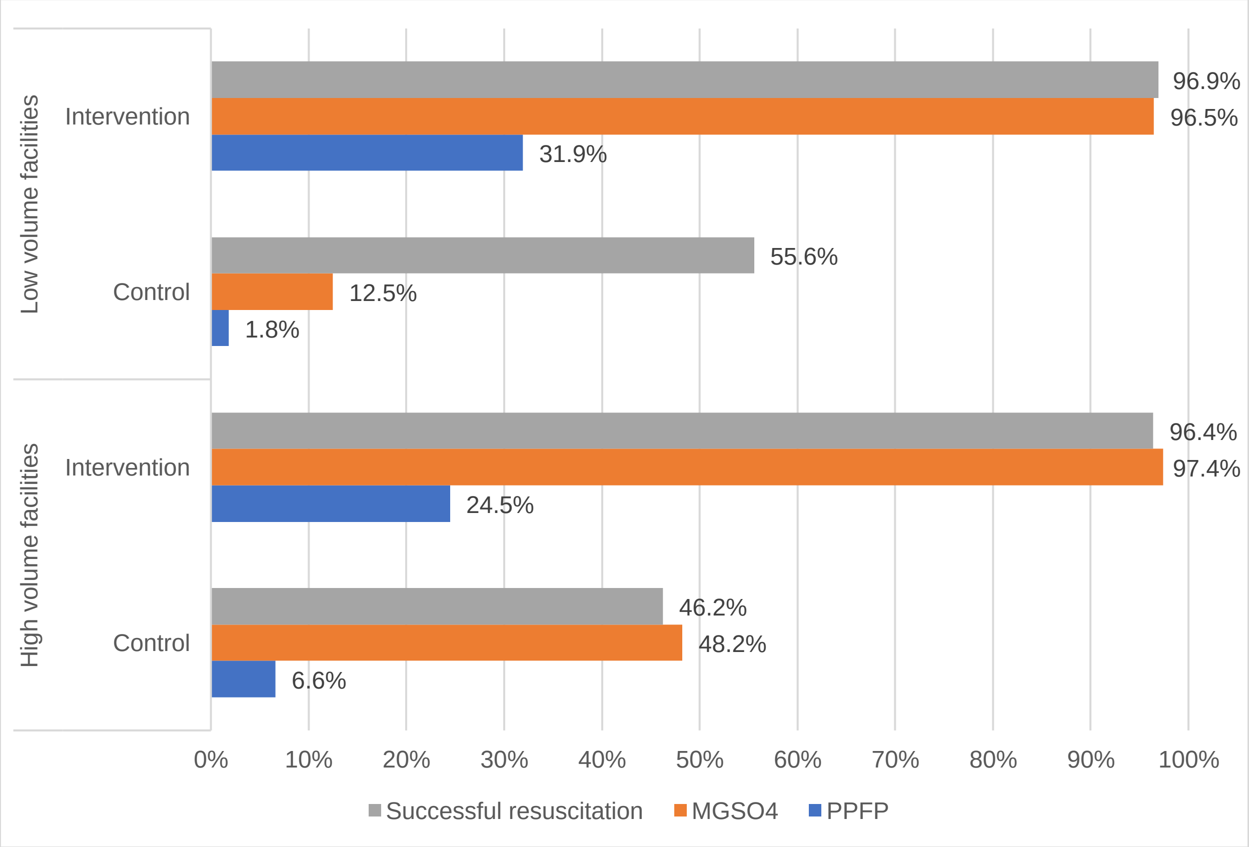

Supplement to: Kikaya V, Katembwe F, Yabili J, et al. Effectiveness of capacity-building and quality improvement interventions to improve day-of-birth care in Kinshasa, Democratic Republic of the Congo. *Glob Health Sci Pract*. 2024;12(1):e2300236. https://doi.org/10.9745/GHSP-D-23-00236

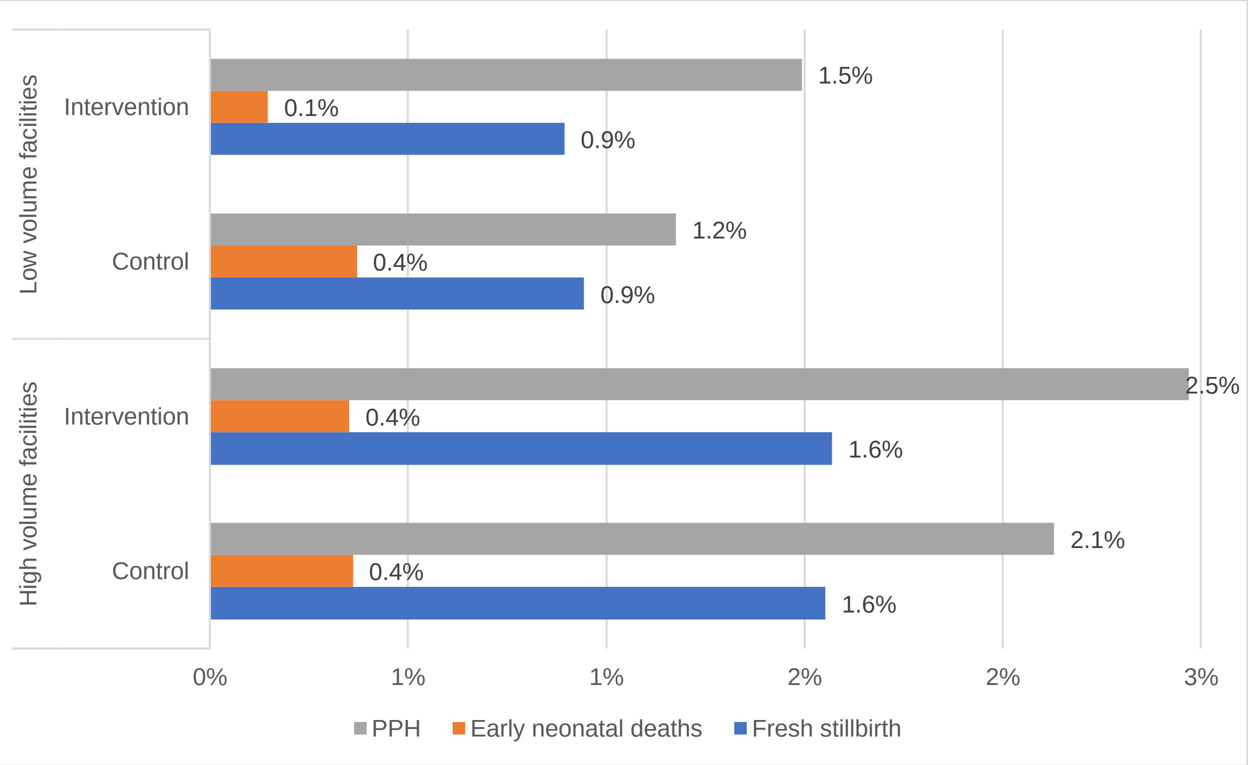

SUPPLEMENT TABLE. Changes in Client Experience and Satisfaction With Care

Subscale	Short scale person-centered maternity care items	May 2018 (n=208)		November 2019 (n=328)	
		No.	%	No.	%
Dignity and respect	Did the doctors, nurses or other health care providers call you by your name?				
	0 No, never	25	12.0	35	10.7
	1 Yes, a few times	43	20.7	21	6.4
	2 Yes, most of the time	46	22.1	32	9.8
	3 Yes, all the time	99	45.2	239	73.1
	Did the doctors, nurses or other staff at the facility treat you with respect?				
	0 No, never	7	3.4	7	2.1
	1 Yes, a few times	41	19.7	16	4.9
	2 Yes, most of the time	60	28.8	56	17.1
	3 Yes, all the time	100	48.1	249	75.9
	Did the doctors, nurses or other staff at the facility treat you in a friendly manner?				
	0 No, never	18	8.7	6	1.8
	1 Yes, a few times	47	22.6	27	8.2
	2 Yes, most of the time	62	29.8	52	15.9
	3 Yes, all the time	81	38.9	243	74.1
	During examinations in the labor room, were you covered up with a cloth or blanket or screened with a curtain so that you did not feel exposed?				
	0 No, never	100	49.8	92	28.6

Supplement to: Kikaya V, Katembwe F, Yabili J, et al. Effectiveness of capacity-building and quality improvement interventions to improve day-of-birth care in Kinshasa, Democratic Republic of the Congo. *Glob Health Sci Pract*. 2024;12(1):e2300236. https://doi.org/10.9745/GHSP-D-23-00236

Subscale	Short scale person-centered maternity care items	May 2018 (n=208)		November 2019 (n=328)	
		No.	%	No.	%
	1 Yes, a few times	22	10.9	14	4.3
	2 Yes, most of the time	6	3.0	20	6.2
	3 Yes, all the time	73	36.3	196	60.9
Communication and autonomy	Did you feel like the doctors, nurses or other staff at the facility involved you in decisions about your care?				
	0 No, never	117	58.5	109	36.3
	1 Yes, a few times	40	20.0	28	9.3
	2 Yes, most of the time	10	5.0	30	10.1
	3 Yes, all the time	33	16.5	133	44.3
	Did the doctors, nurses or other staff at the facility ask your permission/consent before carrying out procedures and examinations?				
	0 No, never	129	60.9	114	36.3
	1 Yes, a few times	30	14.5	35	9.3
	2 Yes, most of the time	19	9.2	43	10.1
	3 Yes, all the time	32	15.5	128	44.3
	During the delivery, do you feel like you were able to be in the position of your choice?				
	0 No, never	195	94.7	221	75.4
	1 Yes, a few times	5	2.4	5	1.8
	2 Yes, most of the time	2	1.0	13	4.4
	3 Yes, all the time	4	1.9	54	18.4
	Did the doctors and nurses explain to you why they were carrying out examinations or procedures?				
	0 No, never	67	32.7	60	19.7
	1 Yes, a few times	39	19.1	27	8.9
	2 Yes, most of the time	29	14.1	40	13.0
	3 Yes, all the time	70	34.1	178	58.4
	Did the doctors and nurses explain to you why they were giving you any medicine?				
	0 No, never	41	20.1	44	13.8
	1 Yes, a few times	33	16.3	19	6.0
	2 Yes, most of the time	31	15.3	35	11.0
	3 Yes, all the time	98	48.3	221	69.2
	Did you feel you could ask the doctors, nurses or other staff at the facility any questions you had?				
	0 No, never	48	23.6	13	4.5
	1 Yes, a few times	45	22.3	19	6.6
	2 Yes, most of the time	22	10.8	39	13.4
	3 Yes, all the time	88	43.3	219	75.5
Supportive care	Did the doctors and nurses at the facility talk to you about how you were feeling? This evaluation was a prospective pre- post- intervention longitudinal time series.				
	0 No, never	59	28.7	31	9.7
	1 Yes, a few times	47	22.8	28	8.8
	2 Yes, most of the time	41	19.9	56	17.5

Supplement to: Kikaya V, Katembwe F, Yabili J, et al. Effectiveness of capacity-building and quality improvement interventions to improve day-of-birth care in Kinshasa, Democratic Republic of the Congo. *Glob Health Sci Pract*. 2024;12(1):e2300236. https://doi.org/10.9745/GHSP-D-23-00236

**Subscale**	**Short scale person-centered maternity care items**	**May 2018 (n=208)**		**November 2019 (n=328)**	
		**No.**	**%**	**No.**	**%**
	3 Yes, all the time	59	28.6	205	64.1
	When you needed help, did you feel the doctors, nurses or other staff at the facility paid attention?				
	0 No, never	27	13.9	13	4.1
	1 Yes, a few times	58	27.9	23	7.2
	2 Yes, most of the time	39	18.8	64	19.9
	3 Yes, all the time	84	40.3	221	68.8
	Did you feel the doctors, nurses or other staff at the facility took the best care of you?				
	0 No, never	7	3.4	2	0.6
	1 Yes, a few times	54	26.1	11	3.4
	2 Yes, most of the time	30	14.5	46	14.1
	3 Yes, all the time	116	56.0	267	81.9
**Mean person-centered maternity care score (Mean, standard deviation [SD])**		19.62	(SD8.67)	28.36	(SD7.57)
